# Effects of chitosan nanoparticles loaded with mesenchymal stem cell conditioned media on gene expression in *Vibrio cholerae* and Caco-2 cells

**DOI:** 10.1038/s41598-022-14057-5

**Published:** 2022-06-13

**Authors:** Masoumeh Saberpour, Shahin Najar-peeraye, Saeed Shams, Bita Bakhshi

**Affiliations:** 1 Department of Bacteriology, Faculty of Medical Sciences, Tarbiat Modares University, Tehran, Iran; 2 Cellular and Molecular Research Center, Qom University of Medical Sciences, Qom, Iran

**Keywords:** Microbiology, Stem cells

## Abstract

*Vibrio* (*V.*) *cholerae* forms a pellicle for self-defense in the pathological conditions in the intestine, which protects it against antibiotics and adverse conditions. Targeting biofilm genes and Toll-like receptors (TLRs) is one of the new strategies to combat multidrug-resistant bacteria. The objective of this study was to evaluate the effect of mesenchymal stem cell conditioned media (MSC CM; 1000 µg), chitosan nanoparticles incorporated with mesenchymal stem cell conditioned media (MSC CM-CS NPs; 1000 µg + 0.05%), and chitosan nanoparticles (CS NPs; 0.05%) on the expression of *bap1* and *rbmC* biofilm genes in *V. cholerae* and *TLR2* and *TLR4* genes in Caco-2 cells. The bacteria were inoculated in the presence or absence of MSC CM, MSC CM-CS NPs, and CS NPs for 24 h at 37 °C to evaluate the expression of biofilm genes. The Caco-2 cells were also exposed to *V. cholerae* for 1 h and then MSC CM, MSC CM-CS NPs, and CS NPs for 18 h at 37 °C. After these times, RNA was extracted from Caco-2 cells and bacteria exposed to the compounds, and the expression of target genes was evaluated using real-time PCR. Caco-2 cell viability was also assessed by MTT assay. After adding MSC CM, MSC CM-CS NPs, and CS NPs to *V. cholerae* medium, the percentage reduction in gene expression of *bap1* was 96, 91, and 39%, and *rbmC* was 93, 92, and 32%, respectively. After adding MSC CM, MSC CM-CS NPs, and CS NPs to the Caco-2 cell medium, the percentage reduction in the gene expression of *TLR4* was 89, 90, and 82%, and *TLR2* was 41, 43, and 32%, respectively. MTT showed that Caco-2 cell viability was high and the compounds had little toxicity on these cells. Finally, it suggests that MSC CM-CS NPs designed may be a therapeutic agent to combat inflammation and biofilm formation in multidrug-resistant *V. cholerae*. However, further studies in vivo are also recommended.

## Introduction

Cholera is a global concern in medical science and is a human health problem worldwide, especially in developing countries such as Iran^1^. The WHO estimated that over 5 million cases suffer from it per year^2^. Although antibiotic therapy is the first step to healing bacterial infections, multidrug-resistance (MDR) strains have led to limited use of antibiotics. Multidrug resistance is prevalent in many bacteria, which may be due to excessive exposure to antibiotics, environmental stress, genetic mutations, and biofilm formation^3,4^. When *V. cholerae* is exposed to antibiotics, a stressful situation develops, and this bacterium forms a biofilm in response to these conditions^5^. Biofilm pellicles are formed from the attachment of bacterial communities to surfaces. The initial step is attachment and then microcolony formation by extrapolymeric substances. Two essential matrix proteins, RbmC and Bap1, are secreted from *V. cholerae* at different times during biofilm formation and play different roles within the biofilm. Bap1 is responsible for binding the biofilm to the surface, while RbmC organizes cell-to-cell connectivity^6^. In previous studies, it is demonstrated that the absence of RbmC does not have a high effect on the structure of the biofilm pellicle in *V. cholerae*. However, a significant loss of pellicle was specified for cells lacking both Bap1 and RbmC^7^.

In addition, cholera is an inflammatory disease of the small intestine. Under normal conditions, innate immune responses contribute to the protection of the gastrointestinal tract epithelium to guarantee the stability of the body environment^8^. One of the most important components of innate immunity that initiates inflammation is Toll-like receptors. Information on the role of TLRs is a critical subject for the discovery of appropriate composites to preserve homeostasis in the gastrointestinal tract. TLRs are the molecular regulators that sense environmental threats and conserve the host from microbial infection. TLRs are pattern recognition receptors (PRRs) that detect internal patterns such as tissue damage (damage-associated molecular patterns or DAMPs), external pathogens (pathogen-associated molecular patterns or PAMPs), and molecular components (microbe-associated molecular patterns or MAMPs)^9^. Usually, they are useless, but after exposure to the patterns derived from various microbes/cells and counteracting TLR2 and TLR4, they can initiate the inflammatory response in the different tissues. After encountering pathogens, including *V. cholerae*, TLRs extend to other intestinal epithelium cells, such as Paneth cells, and they induce innate and adaptive immunity responses^10^. TLR4 and TLR2 signaling by the MyD88‐dependent pathway are important for immune responses since TLR4 connects to lipopolysaccharide (LPS) and TLR2 binds to lipopeptides and peptidoglycans and then activates the inflammatory cytokine cascade consisting of TNF-α, IL-1, and IL-6^11^. It is clear that if inflammation persists and inflammatory responses are not controlled, the disease can develop^12^. Hence, reducing inflammation in the treatment of microbial infections is a crucial subject.

Today, researchers are interested in making nonantibiotic compounds a new therapeutic strategy in the control of bacterial infection^13^. Among them, chitosan and its derivatives have very interesting and non-toxic biological activities, which include anti-microbial, anti-oxidant, anti-cancer, and anti-inflammatory activities^14^. In a study by Siddhardha et al., the anti-biofilm activity of chrysin-encapsulated chitosan nanoparticles (CCNPs) after exposure to *Staphylococcus aureus* was evaluated^15^. The effect of chitosan nanoparticles against *Neisseria gonorrhoeae* was also reported by Alqahtani et al.^16^.

Mesenchymal stem cells (MSCs) can be isolated from various adult tissue and their antibacterial effects have been proven^17,18^. In 2020, Bujňáková and colleagues demonstrated that canine bone marrow mesenchymal stem cell-conditioned media (cBM MSC CM) and may represent an important new approach to managing biofilm formation and Quorum Sensing in bacterial infections^19^. Another study established that MSCs contribute to improving anti-inflammatory activity and increasing angiogenesis. Therefore, MSC therapy may be an effective treatment approach for epithelial injuries^20^.

In general, any low-toxicity drug compounds with anti-inflammatory properties that reduce the expression of these major matrix proteins can lead to a decrease in biofilm formation and have a high effect from available antibiotics. Therefore, targeting biofilm genes and TLRs is one of the novel strategies to combat MDR-*V. cholerae*. We aimed to investigate the expression of the *bap1* and *rbmC* biofilm genes in *V. cholerae* in the presence and absence of three compounds MSC CM, MSC CM-CS NPs, and CS NPs, and also to evaluate the expression of *TLR2* and *TLR4* genes in Caco-2 cells exposed with *V. cholerae* and these compounds.

## Materials and methods

### Bacterial culture and growth conditions

A multidrug-resistant clinical strain of *V. cholerae* stored in the collection of the Bacteriology Department of Tarbiat Modares University, Tehran, Iran, was used. The strain was resistant to tetracycline, ciprofloxacin, chloramphenicol, cotrimoxazole, and trimethoprim. The bacterial culture was performed in 1 mL brain heart infusion (BHI) broth (Merck, Germany) at 37 °C until reaching the log phase. The bacterial suspension concentration was determined by measuring the absorbance at 540 nm and comparing it with the standard 0.5 McFarland optical density (OD). According to the growth curve, the bacterial suspension with an OD of 1:0 (~ 10^8^ CFU/mL) was used^21^.

### Preparations of cells

Bone marrow-derived mesenchymal stem cells (BM-MSCs) and Caco-2 cells were purchased from the Iranian Biological Resource Center and Pasteur Institute of Iran, respectively. BM-MSCs were confirmed by assaying the differentiation of the cells into osteoblasts and adipocytes using an immunohistochemistry (IHC) assay. BM-MSCs were also characterized using the flow cytometry method for CD34, CD45, CD44, and CD73 markers^18^.

### Preparation of MSC CM and nanoparticles

BM-MSCs were cultured in low‐glucose Dulbecco's modified Eagle’s medium (DMEM) (Gibco, USA) supplemented with 10% fetal bovine serum (FBS) (Gibco, USA) and 1% penicillin/streptomycin (Gibco, USA) at 37 °C in a humidified atmosphere containing 5% CO_2._ The medium was replaced after every 2 days. A total of 5 × 10^5^ cells were seeded in a T75 flask (SPL, Korea) containing 15 mL of DMEM supplemented with 10% FBS. When the confluency of the cells was near 90% at passage 2, the medium was replaced with serum-free DMEM. Subsequently, MSC CM was collected and centrifuged at 4000 rpm for 30 min and finally stored at − 80 °C until use^22^. According to our previous study, chitosan nanoparticles were synthesized, characterized, and loaded with the supernatant of mesenchymal stem cells^23^. At all stages of this study, mesenchymal stem cell conditioned media (MSC CM; 1000 µg), chitosan nanoparticles incorporated with mesenchymal stem cell conditioned media (MSC CM-CS NPs; 1000 µg + 0.05%), and chitosan nanoparticles (CS NPs; 0.05%) were used.

### Evaluation of the expression of *bap1* and *rbmC* biofilm genes in *V. cholerae*

*Vibrio cholerae* cell suspensions were inoculated (1:100 dilution) into 1 mL BHI broth medium containing 0.05% sucrose. The bacterial suspension was inoculated with MSC CM, MSC CM-CS NPs, and CS NPs overnight at 37 °C to evaluate the expression of biofilm genes. After this time, each well was washed three times with PBS, and adherent cells were harvested to evaluate the expression of biofilm-related genes. PBS and *V. cholerae* without exposure to the compounds were used as negative and positive controls, respectively. Each assay was performed in triplicate.

### Caco-2 cell culture

Caco-2 cells were cultured in DMEM supplemented with 10% FBS, 1% l glutamine (DNA Biotech, Iran), and 1% penicillin/streptomycin and incubated at 37 °C with 5% CO_2_. The culture medium was changed every two days, and when the confluency reached 80%, the cells were passaged. Since cells in monolayer culture with full confluency can form polarized cells while maintaining cell surface molecules, we explored cells at 85% confluency for all experiments^24^.

### MTT assay

Caco-2 cell viability was estimated by the conventional MTT 3-(4,5-dimethylthiazol-2-yl)-2,5-diphenyl tetrazolium bromide assay. This was tested to evaluate the viability of Caco-2 cells after the exposure time to bacteria, MSC CM, MSC CM-CS NPs, and CS N. Briefly, 2 × ${10}^{4}$ Caco-2 cells per well were seeded into a 96-well plate and cultured for 24 h at 37 °C. The medium was removed, and the cells were exposed to bacterial suspension (10 bacteria per epithelial cell; MOI: 10), MSC CM, MSC CM-CS NPs, and CS NPs separately for 24 h. To assess cytotoxicity, a separate test was also performed for 72 h. After these times, the medium was removed, and MTT solution was added for 3 h at 37 °C. Then, the formazan crystals formed in cells were dissolved in 100 μL of dimethyl sulfoxide (Sigma Aldrich, USA). The resulting purple solution was measured using an ELISA reader (OD 540 nm) (800 TS, BioTek, Winooski, Vermont, USA). Each assay was performed in triplicate^25^.

### Exposure of Caco-2 cells to *V. cholerae*, MSC CM, MSC CM-CS NPs, and CS NPs

For this purpose, five groups were designed: (i) untreated Caco-2 cells (negative control); (ii) Caco-2 cells incubated with *V. cholerae* (MOI: 10-positive control); (iii) Caco-2 cells + *V. cholerae* + MSC CM; (iv) Caco-2 cells + *V. cholerae* + CS NPs; and (v) Caco-2 cells *V. cholerae* + MSC CM-CS NPs. Caco-2 cells were cultured in 96-well microplates until they reached 80% confluence. Before treatment, cells were washed three times with PBS. Then, the cells were infected with *V. cholerae* for 1 h, and the extracellular bacteria and medium was removed and replaced with DMEM-free compounds, including 100 µL of MSC CM, MSC CM-CS NPs, and CS NPs, for 18 h at 37 °C. After this time, the supernatant were removed, and then Caco-2 cells were used for total RNA extraction. Each test was performed in triplicate.

### RNA extraction and cDNA synthesis

Total RNA from bacteria and Caco-2 cells was extracted using an RNA Miniprep Super Kit (Bio Basic, Canada) according to the manufacturer’s recommendations. The RNA was assayed by absorbance at OD260/280. Samples with a ratio of 1.8–2.0 were used for cDNA synthesis using Yekta Tajhiz Azma, Iran. According to the protocol, template RNA (5 µL), random primer (0.5 µL), and DEPC-treated water (7.5 µL) were mixed, centrifuged briefly, and incubated for 5 min at 70 °C. Then, 5× first strand buffer (4 µL), dNTPs (1 µL), RNase 40 U/µL (0.5 µL), and M_MLV (1 µL) were mixed and incubated for 60 min at 42 °C, and the reaction was terminated by heating for 5 min at 70 °C. The cDNA samples were stored at − 20 °C until use in the following experiment.

### Real-time PCR analysis

Conventional SYBR Green-based real-time PCR was used for target gene quantification. Real-time PCR was performed by using 10 μL 5× Real-time PCR Master Mix (Biomake, Houston, TX, USA), 1 μL of each primer (Table 1), 2 μL of cDNA, and 6 μL of distilled water in a total reaction volume of 20 μL in Stratagene Mx3000P real-time PCR system (Stratagene, La Jolla, CA). 16S rRNA was used as an endogenous control to normalize the expression levels of target genes of *V. cholerae.* Beta-actin was also utilized as an internal control to normalize the expression levels in RNA samples from Caco-2 cells. The ΔCT of each sample was measured (CT target–CT reference). We used Caco-2 cells not treated as a calibrator, and the ΔΔCT method was used to determine the difference between treated cells and the control. The fold change of gene expression level was calculated using the comparative CT (2^−ΔΔCT^).

**Table 1:** Primers used in this study.

Primers	Primer sequence (5′–3′)	Reference
16S rRNA-F	GGAAACGATGGCTAATACCG	^6^
16S rRNA-R	GCCCTTACCTCACCAACTAG	
bap1-F	CGCTGGCACACTAAACAAG	
bap1-R	CCATACATTCATACCCAAGAGC	
rbmC-F	CGAGCAATAAGAAAGTGG	
rbmC-R	GCCTTCAACTAACCAAC	
TLR2- F	AACTTACTGGGAAATCCTTAC	^26^
TLR2-R	AAAAATCTCCAGCAGTAAAAT	
TLR4-F	CGAGGAAGAGAAGACACCAGT	
TLR4-R	CATCATCCTCACTGCTTCTGT	
Beta-actin-F	TCCCTGGAGAAGAGCTACG	^27^
Beta-actin-R	TAGTTTCGTGGATGCCACA	

### Statistical analysis

The data were analyzed by using GraphPad Prism version 6 using one-way ANOVA and Bonferroni post hoc test. *P* value < 0.05 was accepted as significant. The results of replications were also evaluated as the mean ± standard deviation (SD).

### Ethics approval

The study was reviewed and approved by the Medical Ethics Committee of Tarbiat Modares University (Code: IR.MODARES.REC.1398.060). All methods were also carried out in accordance with the guidelines and regulations related to the committee.

## Results

### Characterization of nanoparticles and in vitro release assay

**Figure 1:**
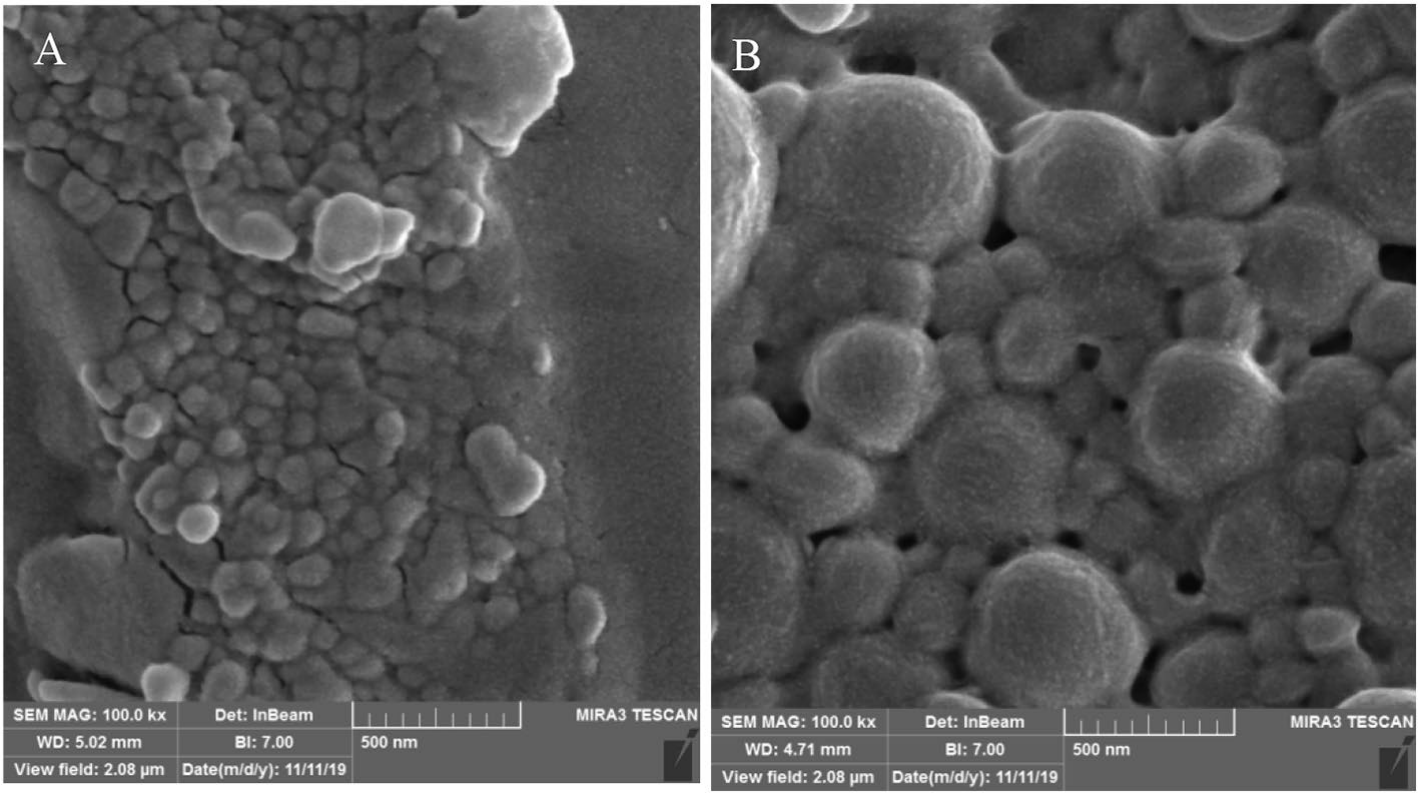
Scanning electron microscope image. CS NPs (**A**) and MSC CM-CS NPs (**B**).

The dispersion of CS NPs size was ~ 414 nm and the electric charge was 6.90 mV. The scanning electron microscope of the CS NPs showed that they were spherical in shape (Fig. 1A) and NPs were cross-linked with MSC CM (Fig. 1B). Entrapment efficiency (EE) of MSC CM-CS NPs was 77%, indicating that acceptable amounts of MSC CM were loaded on CS NPs. In addition, at pH = 7.2 and after 72 h, the release rate of MSC CM from MSC CM-CS NPs was 72%.

### Reducing the expression of *bap1* and *rbmC* biofilm genes in *V. cholerae* by MSC CM, MSC CM-CS NPs, and CS NPs

**Figure 2:**
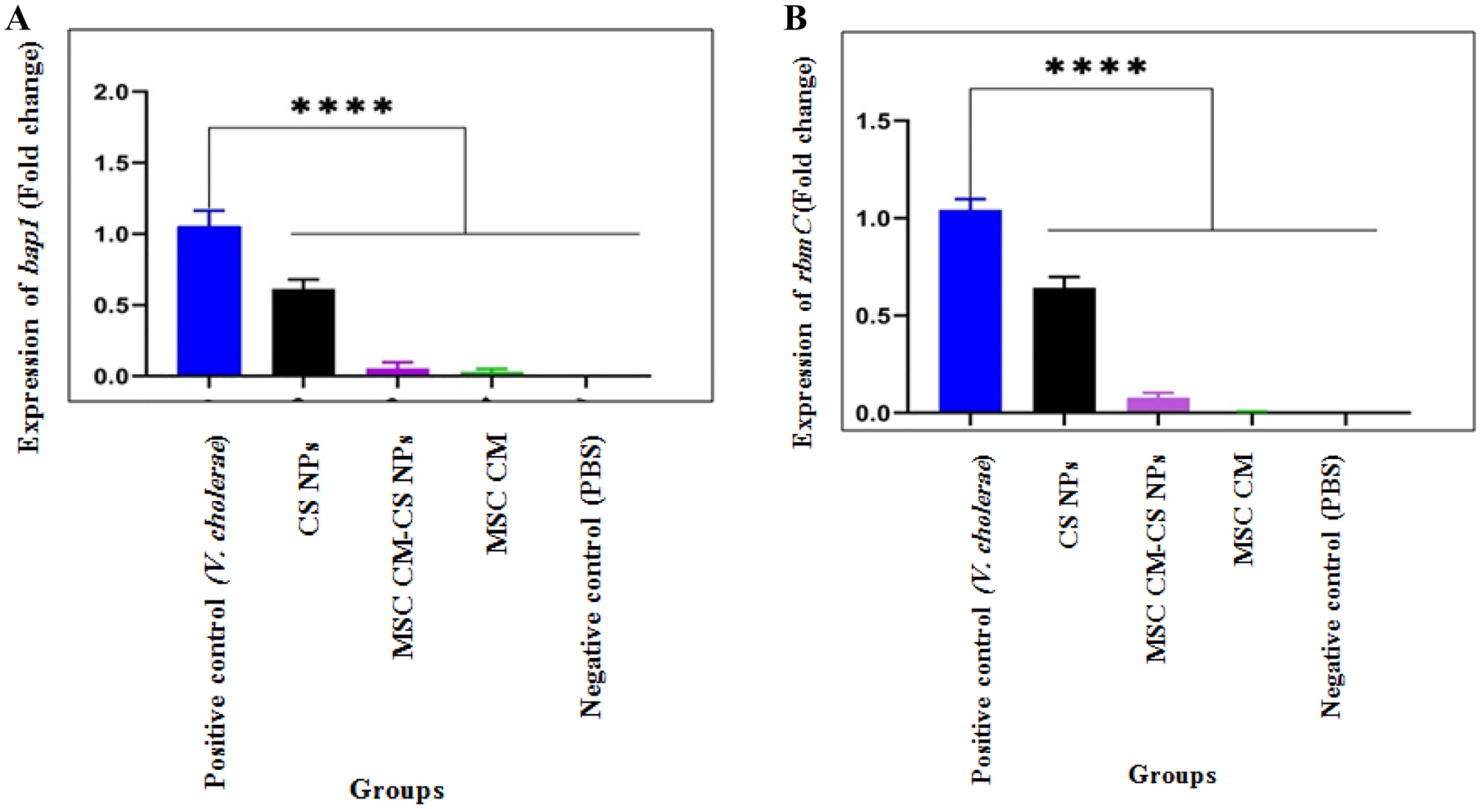
The inhibitory effect of three compounds MSC CM, MSC CM-CS NPs, and CS NPs on *bap1* (**A**) and *rbmC* (**B**) gene expression in *V. cholerae*. PBS and *V. cholerae* without exposure to the compounds were used as negative and positive controls, respectively. Bars are presented as the mean of triplicate experiments. ****p < 0.0001 for *V. cholerae* vs. other groups.

The expression of genes that are responsible for the production of two matrix proteins (Bap1 and RbmC) was compared in the presence and absence of three compounds after 24 h of exposure. After adding MSC CM, MSC CM-CS NPs, and CS NPs to *V. cholerae* culture medium, the percentage reduction in *bap1* was 96, 91, and 39%, and in *rbmC* was 93, 92, and 32%, respectively. The downregulation of the genes encoding matrix proteins, including Bap1 and RbmC, was evident in the presence of MSC CM, MSC CM-CS NPs, and CS NPs. However, the inhibitory effect of MSC CM and MSC CM-CS NPs on *bap1* and *rbmC* expression was greater than that of CS NPs. No significant difference was observed between MSC CM and MSC CM-CS NPs. Also, there was a significant difference in *bap1* and *rbmC* gene expression between different groups in comparison to the *V. cholerae* group (*p* < 0.0001) (Fig. 2A,B).

### Cell viability of Caco-2 cells after treatment with *V. cholerae* and different compounds

**Figure 3:**
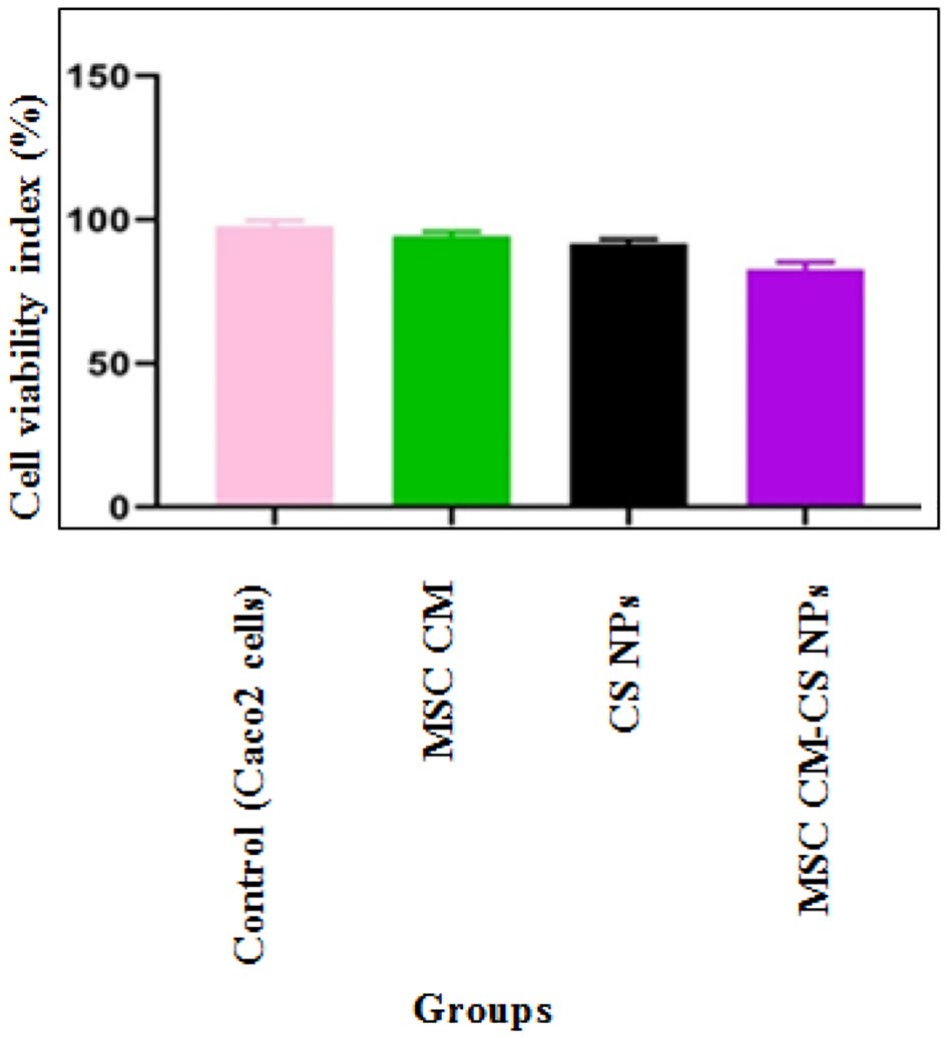
Cell viability of Caco-2 cells treated with MSC CM, MSC CM-CS NPs, and CS NPs after 24 h. Bars are presented as the mean of triplicate experiments. The results were almost similar for all compounds.

The viability of cells was measured as the percentage of live cells relative to the control cells. We found the best results at an MOI of 10; at this MOI, 80–90% of the Caco-2 cells were infected, and the cell viability was > 80% at 24 h. In addition, it was shown that the viability of Caco-2 cells after exposure to the three compounds was the best concentration of MSC CM, MSC CM-CS NPs, and CS NPs, and for 24 h exposure times, with cell viabilities of 80, 79, and 78%, respectively. Also, after 72 h exposure times, cell viabilities were 66.24, 63.93, and 57.3%, respectively. The results were almost similar for all groups. However, the cell persistence against MSC CM was slightly better than that of the other groups (Fig. 3).

### The expression of *TLR2* and *TLR4* genes decreased after exposure to MSC CM, MSC CM-CS NPs, and CS NPs

**Figure 4:**
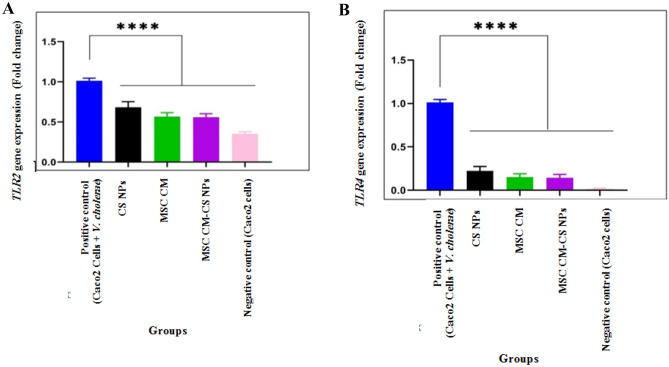
*TLR2* (**A**) and *TLR4* (**B**) gene expression in Caco-2 cells after exposure to *V. cholerae* and then three compounds MSC CM, MSC CM-CS NPs, and CS NPs. Bars are presented as the mean of triplicate experiments. ****p < 0.0001 for positive control (Caco-2 cells + *V. cholerae*) vs. other groups.

*TLR2* and *TLR4* gene expression was evaluated at the Caco-2 cells. These expressions decreased after treatment with *V. cholerae* and different compounds. Exposure of Caco-2 cells to *V. cholerae* significantly enhanced *TLR2* and *TLR4* mRNA levels in these cells, while control cells (Caco-2 cells without exposure to *V. cholerae*) did not show significant expression. After adding different compounds, including MSC CM, MSC CM-CS NPs, and CS NPs, to Caco-2 cell medium, the percentage reduction of *TLR2* gene expression was 41, 43, and 32%, and the percentage reduction of *TLR4* gene expression was 89, 90, and 82%, respectively. In addition, there was a significant difference between *TLR2* and *TLR4* gene expression after treatment with *V. cholerae* compared to the cells treated with other compounds (*p* < 0.0001). Also, MSC CM incorporated with CS NPs and MSC CM had a greater inhibitory effect than CS NPs on reducing *TLR2* and *TLR4* gene expression (Fig. 4A,B).

## Discussion

In recent years, the emergence of drug-resistant bacteria has been a public health concern in developing regions^28–30^. Excessive prescription of available antibiotics led to a mutation in bacteria and was conducive to the expansion of multidrug resistance in the bacteria, especially *V. cholerae* strains. *V. cholerae* forms a biofilm pellicle after being exposed to environmental stress, which protects them against antibiotics and adverse conditions These also construct the biofilm membrane as a procedure to survive during the intestinal phases of their life cycle, thus they are protected from environmental stress such as acidic stomach conditions by biofilm formation^23^.

Because bacteria become rapidly resistant to antimicrobial agents, scientists are progressively affording to develop nonantibiotic and anti-biofilm agents with anti-inflammatory properties for therapeutic goals^31^. The application of nanotechnology and mesenchymal stem cells in medical science is popular due to their ability to enhance the bioavailability and biosorption of numerous drugs. Bone marrow-derived mesenchymal stem cells are a crucial component of the innate immune system due to secreted products such as antimicrobial peptides (AMPs) consisting of cathelicidin, LL37, and beta-defensins. A study by Krasnodembskaya et al. showed that the treatment of MSCs with bacteria leads to increased AMP production^32^. Bahroudi et al. reported that BM-MSCs without exposure to *V. cholerae* secreted conditioned media containing soluble proteins and vesicles, displaying significant antimicrobial and anti-biofilm activities against *V. cholerae* strains^18^.

Chitosan nanostructures have various properties, including appropriate carrier, anti-inflammatory, and anti-biofilm activities^33^. In addition, these nanoparticles have a variety of inhibitory effects on the bacteria by neutralizing LPS, inhibiting protein synthesis and RNA, and eliminating essential metals for bacterial growth^34^. In a study, the anti-biofilm activity of chitosan nanoparticles (CSNPs) along with oxacillin (Oxa) and deoxyribonuclease I (CSNP-DNase-Oxa) was evaluated against *Staphylococcus aureus*. The results showed that CSNP-DNase-Oxa has greater anti-biofilm activity than Oxa-loaded nanoparticles without DNase (CSNP-Oxa) and free Oxa (Oxa and Oxa + DNase) under environmental conditions^35^. In another study, the use of chitosan nanoparticles as an effective antimicrobial agent against *Klebsiella pneumoniae, Escherichia coli, Staphylococcus aureus*, and *Pseudomonas aeruginosa* with anti-biofilm activity was also identified^36^.

In a prior study, we evaluated the anti-biofilm effect of MSC CM, MSC CM-CS NPs, and CS NPs on biofilm formation by using the crystal violet method. Our results showed that MSC CM-CS NPs inhibited biofilm formation^23^. Therefore, the present study was designed based on a previous study to determine the inhibitory effects of these compounds on the expression of *bap1* and *rbmC* biofilm genes in *V. cholerae* and *TLR2* and *TLR4* genes in Caco-2 cells.

In this study, the findings indicated that MSC CM, MSC CM-CS NPs, and CS NPs reduced biofilm formation. MSC CM alone has the best anti-biofilm effect against *V. cholerae*, and the combination of MSC CM with chitosan nanoparticles does not have a synergistic effect against the biofilm formation of *V. cholerae*. Although there was no significant difference between the two compounds, MSC CM and MSC CM-CS NPs, the release of MSC CM from the nanostructures was slow (72% after 72 h) and therefore could have a better and more stable effect. Therefore, MSC CM-CS NPs can be used as new therapeutic strategies against multidrug-resistant *V. cholerae* strains. However, the mechanism of the effect of MSC CM combined with CS NPs is unknown. It seems that supernatant-derived soluble proteins have disruptive effects on the cell wall; they enhance CS NPs importation into the cytoplasm and lead to inhibition of mRNA and protein synthesis by chitosan. Thus, the expression of biofilm formation genes in *V. cholerae*, such as *bap1* and *rbmC*, is reduced.

Normally, TLR4 and TLR2 bind to different bacterial antigens, and lead to the production of inflammatory cytokines^37^. After the binding of *V. cholerae* to small intestinal receptors such as TLR2 and TLR4, the cytokine cascade is activated, followed by inflammation^38^. In addition, *V. cholerae* can directly induce a cascade of chemokines that absorb different inflammatory cells^39^. In one study, *V. cholerae* induced an inflammatory response relative to the TLR and nucleotide-binding oligomerization domain, NOD, which helps to design suitable drugs for specific targets and regulation of inflammation^40^.

Our findings showed that MSC CM, MSC CM-CS NPs, and CS NPs are effective composites for decreasing *TLR2* and *TLR4* gene expression in Caco-2 cells. However, the decrease in *TLR2* and *TLR4* gene expression was greater in the presence of MSC CM and MSC CM-CS NPs than in CS NPs. These findings are consistent with some of other studies. For example, in a study, it was demonstrated that MSC CM modulates cytokine expression in macrophages, which leads to TLR7/8 stimulation^41^. Another study conducted by Démoulins et al., the results demonstrated that alginate-coated chitosan modulated TLR2 on blood dendritic cells^42^.

## Conclusion

In conclusion, MSC CM formulated in MSC CM-CS NPs with low cytotoxicity has long-lasting anti-biofilm effects against MDR*-V. cholerae* strain. The designed nanocomposite also demonstrated a significant impact against immune responses related to TLR2 and TLR4*.* Therefore, the compound might be considered anti-biofilm nanocomposite to inhibit biofilm pellicle formation and cytokine cascades that are produced after activating TLR2 and TLR4 and initiate after exposure to *V. cholerae* in the human body environment. In future studies, evaluation of the inhibitory effect of MSC CM, MSC CM-CS NPs, and CS NPs on other genes of *V. cholerae* biofilm formation and other inflammatory genes is recommended. Further research in the animal model is also needed to investigate in vivo conditions, evaluate inflammatory cytokines, and better understand the mechanisms of action of the compounds studied.

### Limitations

It was not possible to measure cytokines due to lack of adequate funding, and this was one of the limitations of the study at this stage. Also, due to financial constraints, we could not examine the structure of the biofilm using an electron microscope.

## Data Availability

The datasets used and/or analyzed during the current study are available from the corresponding author on reasonable request. In this study, no data deposition was done about the expression of target genes.

## Abbreviations

MSC CM-CS NPs: Chitosan nanoparticles incorporated with mesenchymal stem cell-conditioned media
MSC CM: Mesenchymal stem cell conditioned media
*V. cholerae*: *Vibrio cholerae*
MDR: Multidrug resistance
CS NPs: Chitosan nanoparticles
AMPs: Antimicrobial peptides
TLRs: Toll-like receptors
DAMPs: Damage-associated molecular patterns
PAMPs: Pathogen-associated molecular patterns
MAMPs: Microbe-associated molecular patterns
FBS: Fetal bovine serum
DMEM: Dulbecco's modified Eagle’s medium
PBS: Phosphate-buffered saline
cDNA: Complementary DNA
dNTPs: Deoxynucleoside triphosphates
16S rRNA: 16S ribosomal RNA
ELISA: Enzyme-linked immunosorbent assay
PRRs: Pattern recognition receptors
